# Multi-Bit Biomemory Based on Chitosan: Graphene Oxide Nanocomposite with Wrinkled Surface

**DOI:** 10.3390/mi11060580

**Published:** 2020-06-10

**Authors:** Lei Li, Guangming Li

**Affiliations:** 1 Key Laboratory of Functional Inorganic Material Chemistry (MOE), School of Chemistry and Materials Science, Heilongjiang University, Harbin 150080, China; lileidtk@hlju.edu.cn; 2 HLJ Province Key Laboratories of Senior-Education for Electronic Engineering, Heilongjiang University, Harbin 150080, China

**Keywords:** multi-bit biomemory, tristability, CS:GO nanocomposites, wrinkled surface biomaterial

## Abstract

Chitosan (CS) is one of the commonly affluent polysaccharides that are attractive biomaterials as they are easily found in different organisms and are biocompatible. An environment-friendly multi-bit biomemory was successfully achieved on the basis of CS as a favorable candidate for resistive-switching memory applications. By incorporating graphene oxide (GO) into CS, the multi-bit biomemory device (indium tin oxide (ITO)/CS:GO/Ni) was obtained through the solution-processable method, which had a high current ratio among a high, intermediate, and low resistance state as well as a low SET/RESET voltage. GO acting as trapping sites in the active layer might be responsible for the biomemory mechanism. This research opens up a new avenue towards renewable and environmentally benign CS-based materials for biodegradable electronic devices.

## 1. Introduction

Resistive-switching memory devices, originated from the beneficial properties like high density, large scalability, low power consumption, and high endurance and retention performance, have emerged as promising candidates for future high-performance nonvolatile data memory [1,2]. They mostly perform binary digital data storage by encoding “0” and “1” as a high and low resistance state (HRS and LRS) in response to an applied external electric voltage, substituted for flash memory and dynamic random access memory (DRAM). Nevertheless, the memory capacity on the basis of electrical bistability is limited at 2^n^. A promising method to enhance the data storage capacity is by promoting the number of conductive states in memory devices to 3 or higher [3,4]. For next-generation ultrahigh density and high-speed biomemory, multi-bit resistive-switching biomemory devices can be seen as promising building blocks.

Si-based electronic devices have dramatically extended approaches in the domain of high-speed computers, high-efficiency photovoltaic cells, and portable consumer electronics [5,6,7,8,9]. As their operating lifetime gradually becomes shorter, not only a rapid depletion of natural and nonrenewable resources, but also a generation of large amounts of electronic waste with toxic or nondegradable materials has become ubiquitous nowadays. Hence, there is a great demand of sustainable biomaterial electronics. Biological polymers offer novel opportunities for nonvolatile memory by virtue of their biocompatibility, environmental benignity together with sustainability [10,11,12,13]. Biomaterial-based passive or active components in biocompatible and biodegradable devices revolutionize state-of-the-art electronic devices and systems that currently have counted on conventional semiconductor technologies [14,15].

Chitosan (CS), obtained from deacetylation of chitin, is the second most abundant element after cellulose. Its hydrophilic, biocompatible, moldable, biodegradable, nontoxic, cheap, good absorbent, and renewable properties contribute to applications in medicine, pharmaceutical industry, food production, plant growing, and chemical engineering [16]. Graphene oxide (GO), with functional groups on its basal planes and edges, is a layered material consisting of hydrophilic oxygenated graphene sheets. Apart from that, the amino and hydroxyl groups in CS are treated as reaction sites to GO in adsorption studies and biosensor applications [17,18]. This contribution was to prepare CS:GO nanocomposite films by a simple spin casting method to avoid sophisticated instruments or vacuum filtration techniques. Amino groups of CS become polyelectrolytic by carrying R-NH^3+^ in the solution, which result in a cathodic biopolymer [19]. CS, however, is a poor conductor [20].

Biomemory devices on account of biocompatible materials, such as ferritin, cellulose, and DNA, typically exhibit binary resistive-switching behavior [21]. Multi-bit biomemory behaviors based on CS, however, have never been reported [19,22]. Herein, a multi-bit biomemory device (indium tin oxide (ITO)/CS:GO/Ni) was first successfully manufactured. Compared to the binary device with the memory capacity of 2^n^, the ternary biomemory capacity can reach up to 3^n^. The multi-bit memory performance based on CS:GO nanocomposites was achieved by varying chemical component ratios. The following reasons suggest that our choice (CS:GO nanocomposites) for biomemory behaviors is feasible. (1) CS is an insulator in the native state. An appropriate amount of GO in the CS matrix can play a role in charge-trap elements to tune the conductivity of the film. (2) Abundant carbonyl and hydroxyl groups in carboxylated CS qualifies it to be water-soluble, which then allows for easy device processing. (3) Existence of amino and hydroxyl groups in CS can absorb GO, which will facilitate the charge transfer. (4) CS can be easily processed as uniform and stable films under normal conditions in air. Apart from a simple fabrication process, the proposed device features outstanding resistive-switching properties and reliability. As for biomemory materials, CS:GO nanocomposites are more advantageous for multi-bit data storage. Advances render CS:GO nanocomposites suitable for applications in next-generation high-density green data-storage devices and biointegrated electronics.

## 2. Materials and Methods

Multilayers of GO (black powder) were purchased from Tanfeng Tech. Inc. (Suzhou, China) when carboxylated CS (*M*_n_ = 48 kg/mol and a degree of deacetylation = 90 mol%) with molecular formula (C_6_H_11_NO_4_)_n_ was purchased from Aladdin (Tianjin, China). For the manufacture of biomemory devices, CS:GO nanocomposite solutions (5 mg/mL) with a concocting mass ratio between chemical components (2:1, 5:1, and 10:1, *w*/*w*) were homogeneously prepared in deionized water by ultrasonic vibration for 24 h. The substrates were precleaned by a sonication-aided washing process that included the following steps: acetone for 1 h → absolute alcohol for 1 h → deionized water for 40 min → drying in an oven at 40 °C for 30 min. At ambient temperature, biomemory devices were fabricated by subsequent steps. The nanocomposite solution was spun onto the precleaned glass slide coated with an ITO layer (the square resistance *R*_sq_ ≤ 6 Ω/sq, 185 nm thick, transmittance ≥84%), during which the spin casting method was employed at a speed of 5000 rpm for 60 s. The spun films were dried on a hot plate at 60 °C overnight to remove the residuals of deionized water. The thickness of the obtained CS:GO nanocomposite films (~30 nm) was determined by NanoMap 500LS (3D Profilometer aep Technology, Santa Clara, CA, USA). With a shadow mask, Ni electrodes were eventually deposited onto nanocomposite films (coated on the substrates) by means of thermal evaporation at pressure approaching 10^−5^ Torr. The top metal electrodes were determined to have a thickness of 200 nm and a size of 1.0 × 1.0 mm^2^.

Techniques like scanning electron microscopy (SEM), fourier transform infrared (FTIR) spectroscopy, Raman spectroscopy, and thermogravimetric analyses (TGA) were implemented to characterize the morphological, structure, and thermal properties of CS:GO nanocomposites. Both morphological and cross-sectional profiles of the CS:GO nanocomposite films were implemented by Apreo Scanning Electron Microscope (Themoscientific; Waltham, MA, USA). FTIR spectroscopy (Foss DS 2500 Infrared Spectrometer; Munkedal, Sweden) using KBr pellets were carried out, sweeping from 400 cm^−1^ to 4000 cm^−1^. Raman spectroscopy (Horiba Jobin Yvon; Villeneuve-d’Ascq, France) equipped with a 532 nm laser was employed to achieve the structure information of CS:GO nanocomposites, scanning from 100 cm^−1^ to 3200 cm^−1^. Furthermore, TGA (TA Instruments; New Castle, DE, USA) was utilized to analyze thermal properties of CS:GO nanocomposites at a heating rate of 10 °C/min under N_2_ of 100 mL/min. X-ray Diffraction (XRD) of GO powder was tested by X’Pert PRO XRD meter (Panalytical, Almelo, Finland) with Cu Kα radiation (wavelength = 1.540598 Å). The scan was taken in the 2θ range of 5°–30° with a scan step size of 0.0131303°/mm. HR-TEM images for GO were characterized by a JEM-2100 TEM (JOEL; Tokyo, Japan) operated at 200 kV. The electrical measurements were fulfilled by a semiconductor parameter analyzer (Keithley 4200SCS; Solon, OH, USA). All electrical experiments were conducted without any device encapsulation. The device was initially scanned from 0 V to 6 V (1st sweep) and then scanned once more (2nd sweep), which was followed by a reverse scan from 0 V to −6 V (3rd sweep). Next, the succeeding step repeated it (4th sweep). The top Ni electrode was grounded as the bias voltage was applied to the bottom ITO electrode.

## 3. Results

### 3.1. Characterization of CS:GO Nanocomposites

**Figure 1:**
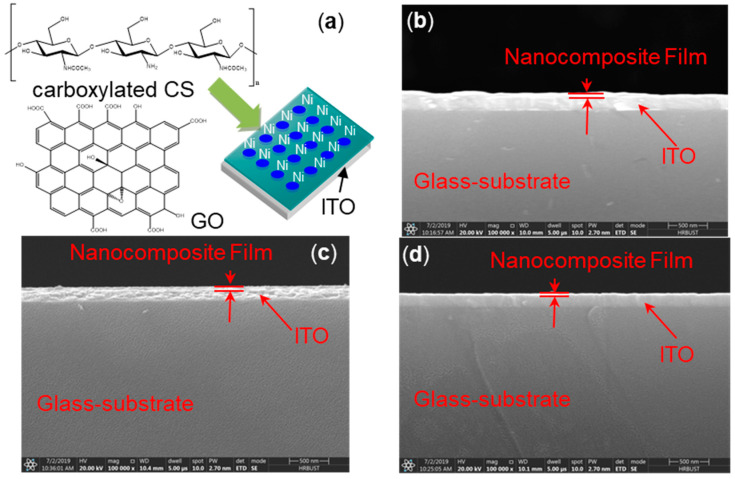
(**a**) Chemical structure of carboxylated chitosan (CS) and graphene oxide (GO) and sandwiched structure of ITO/CS:GO/Ni (right); (**b**–**d**) the cross-sectional profiles of CS:GO nanocomposite films coated on the ITO/glass substrates with a mass ratio of the chemical component CS:GO 2:1, 5:1, and 10:1, respectively.

**Figure 2:**
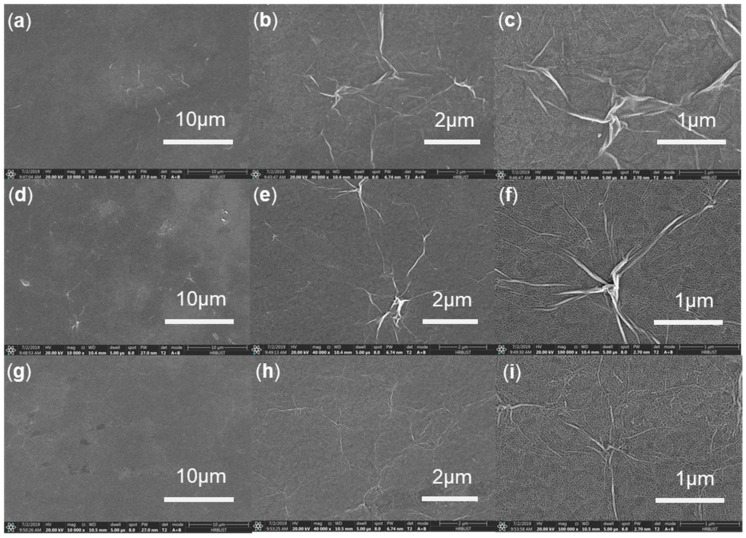
Scanning electron microscopy (SEM) images of the morphology for CS:GO nanocomposite films with the chemical component ratio (**a**–**c**) 2:1, (**d**–**f**) 5:1, and (**g**–**i**) 10:1 *w*/*w*, respectively, in terms of distinct magnification 10,000×, 40,000×, and 100,000×, respectively. (HV: 20 kV, WD: 10.4 mm, dWell: 5 μs).

**Figure 3:**
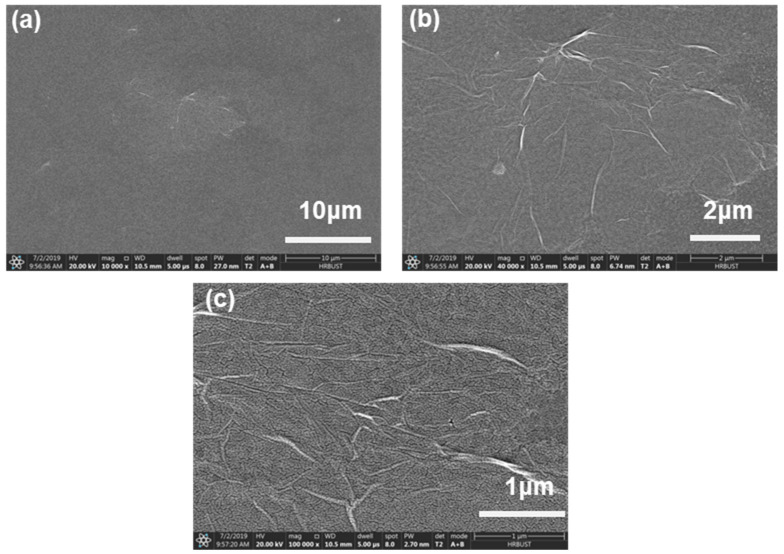
(**a**–**c**) SEM images of the morphological GO film in terms of distinct magnification 10,000×, 40,000×, and 100,000×, respectively. (HV: 20 kV, WD: 10.4 mm, dWell: 5 μs).

As exhibited in Figure 1a, a CS:GO nanocomposite film with ternary biomemory performance is sandwiched between an ITO bottom electrode and a Ni top electrode (i.e., ITO/CS:GO/Ni). The cross-sectional profiles of these three CS:GO nanocomposite films with a distinct weight ratio of 2:1, 5:1, and 10:1 (*w*/*w*), respectively, present a layered structure of GO (Figure 1b–d) with a thickness of roughly 30 nm. The reason for better compatibility between CS and GO is that amino and carboxyl groups in CS can be used to form hydrogen bonding with a variety of oxygen-containing groups in GO and simultaneously inhibit the aggregation of GO [23,24,25,26]. The morphological observation (Figure 2) without agglomeration illustrates that more content of GO brings about more wrinkled nanocomposite film. The strong p–p stacking within GO is responsible for it [25,26]. It indicates that GO is well dispersed in CS, which is beneficial for the use of wrinkled surfaces for this application. For further insight into the wrinkled surface of CS:GO nanocomposite films, the GO aqueous solution (5 mg/mL) was spin-coated onto the glass substrate with ITO layer for which the SEM images of the GO film (Figure 3) were employed to confirm that GO was responsible for the wrinkled surface of CS:GO films.

**Figure 4:**
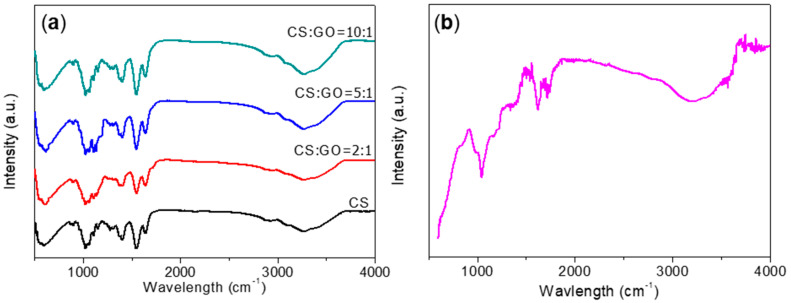
Fourier transform infrared (FTIR) spectra for (**a**) CS and CS:GO nanocomposites, (**b**) GO powder.

FTIR spectroscopy was applied to examine the chemical composition of GO, CS, and CS:GO nanocomposites. The spectra of CS and its blends were similar since they were polysaccharides (Figure 4). The characteristic peaks of pure CS included a N–H bending peak at 1590–1660 cm^−1^, a bridge−O stretching peak at 1154 cm^−1^, and a C–O stretching peak at 1032–1094 cm^−1^. The vibration of the OH group facilitated the peak at 3264 cm^−1^ when giving rise to the peak at 3270 cm^−1^ for all CS:GO nanocomposites. Moreover, the peak at 1152 cm^−1^ stemmed from the vibration of the saccharide structures that caused the peak at 1136 cm^−1^, 1135 cm^−1^, and 1152 cm^−1^ for CS:GO nanocomposites with 2:1, 5:1, and 10:1 (*w*/*w*), respectively. Observed from the FTIR spectrum of GO, GO had a variety of hydrophilic oxygen groups [27]. Among them, the peak at 3348 cm^−^^1^ was attributed to the asymmetric telescopic vibration peak of the hydroxyl (O–H) when the telescopic vibration of the carboxyl group (–COOH) brought about the peak at 1716 cm^−^^1^. The stretching vibration of the undamaged carbon–carbon double bond (C=C) was attributed to the peak at 1621 cm^−^^1^. These results suggested that both CS and GO could be spun together by this mixing system.

**Figure 5:**
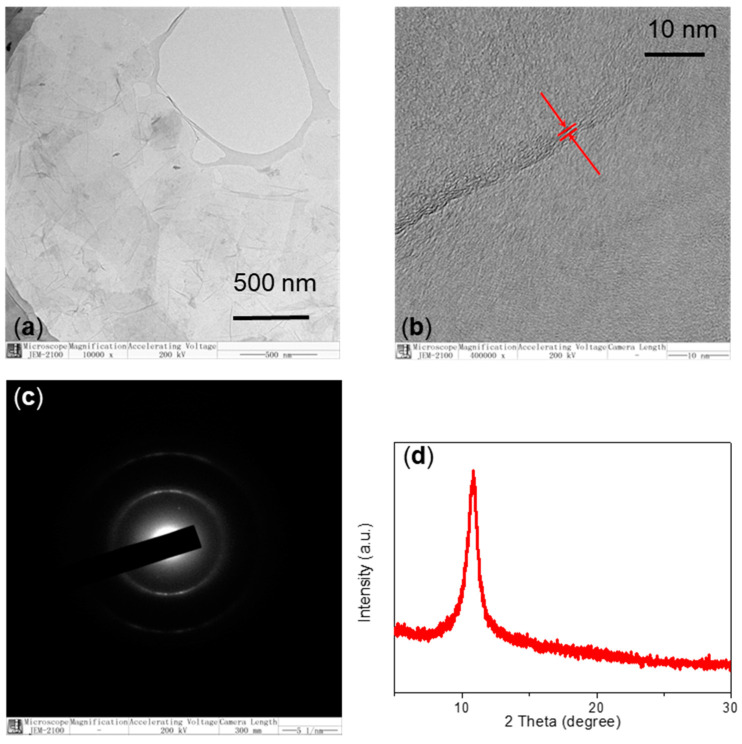
(**a**) Transmission electron microscope (TEM) image of GO powder and (**b**) its high-resolution TEM image; (**c**) selected area electron diffraction (SAED) pattern and TEM micrograph in magnification of 10,000× of GO. (**d**) XRD pattern of GO.

For a characterization of the layered structure, TEM, XRD together with Raman spectroscopy were performed. HR-TEM micrographs showed a two-dimensional (2D) multilayer structure of GO, demonstrated in Figure 5a,b. The selected area electron diffraction (SAED) pattern obtained from TEM is shown in Figure 5c. The formed diffuse ring pattern showed that GO was amorphous [23]. XRD pattern of GO (Figure 5d) showed the dominant peak at 10.8° with interlayer spacing (0.82 nm) larger than pristine graphite (0.34 nm) owing to the addition of oxygen with functional groups [24]. The oxidation degree of GO could be calculated based on the XRD pattern [23,24].

**Figure 6:**
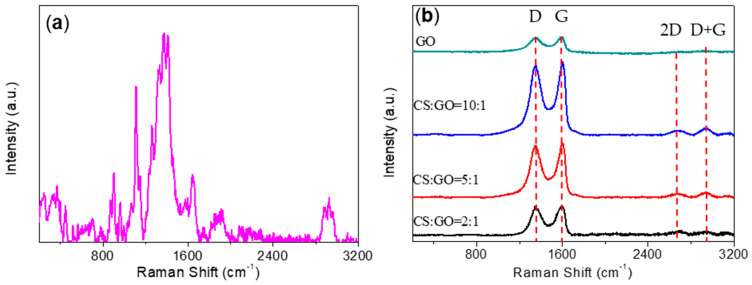
Raman spectra for (**a**) CS, (**b**) GO powder, and CS:GO nanocomposites.

For the Raman spectrum of CS (Figure 6a), the evaluation of CS predominately indicated peaks of symmetric stretches of the C–O–C bond in the 850–900 cm^−1^ region owing to glyosidic bonds while antisymmetric stretches of the C–O–C bond were identified in the 1060–1150 cm^−1^ region. The peak at 1265 cm^−1^ indicated the stretch of the C−OH bond as the one at 1378 cm^−1^ resulted from symmetrical CH_3_. Furthermore, the peak related to the symmetric bond of CH_2_ was exhibited at 1459 cm^−1^. The structural properties of GO and CS:GO nanocomposites were investigated through Raman spectra as well (Figure 6b). All of the samples displayed the distinctive D band (~1350 cm^−1^) and G band (~1590 cm^−1^), demonstrated in Table 1. The intensity ratio of D band to G band (*I*_D_/*I*_G_) was in inverse proportion to the graphitization degree [28,29]. Compared with GO (*I*_D_/*I*_G_ = 0.923), all the nanocomposites had higher *I*_D_/*I*_G_ values, which suggested that more defects were created in CS:GO nanocomposites [30]. In the Raman spectra of GO and CS:GO nanocomposites, the 2D and D + G Raman bands from 2690 cm^−^^1^ to 2950 cm^−^^1^ were observed for all samples. It implied the presence of multilayers [31]. The 2D band was very sensitive to the stacking order of GO along the *c*-axis, and thus, functioned to evaluate the structural parameters of *c*-axis orientation [23]. The D + G band was only observed in samples with significant amounts of defects (trap sites) [32], which arose in all CS:GO nanocomposite samples. Table 1 represents the graph change in Raman intensity ratio (*I*_2D_/*I*_G_) against samples as well. CS:GO nanocomposites showed a higher graphene stacking as observed in the growth of the 2D band (*I*_2D_/*I*_G_ was proportional to the number of graphene layers) [30].

**Figure 7:**
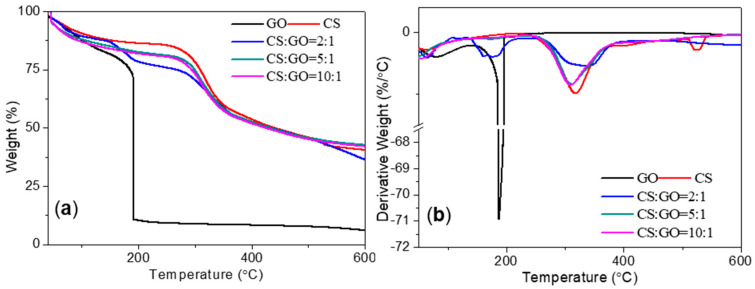
(**a**) Thermogravimetric analyses (TGA) and (**b**) derivative thermogravimetry (DTG) for GO, CS, and CS:GO nanocomposites.

TGA and derivative thermogravimetry (DTG) of GO and CS:GO nanocomposites in N_2_ atmosphere were carried out to study their thermal stability (Figure 7). The weight loss of GO started below 100 °C upon heating, and the major weight loss of GO occurred (60%) at ~200 °C, since oxygen-containing functional groups were pyrolyzed and released gases which resulted in rapid thermal expansion of GO [33]. The TGA plot for GO revealed that there was a small residual weight of 6% after heating to 600 °C under N_2_ atmosphere. TGA and DTG curves for CS:GO nanocomposites were similar to that of CS with three main decomposition stages. The first stage of thermal decomposition started from 44 °C to 287 °C. This stage was originated from physically adsorbed water surface of the polymer. With a maximum peak at 318 °C in the DTG curve, the second stage occurred between 287 °C and 400 °C, and bore the final mass loss of 59.5%. It was bound up with the thermal degradation of the ether bond. Furthermore, the third stage occurred between 400 °C and 600 °C, which might be correlated with the degradation of volatile products of the material. It evidenced the good dispersion of GO in the CS films, which corresponded with the characterization of SEM, FTIR, and Raman spectroscopy.

### 3.2. Multi-Bit Biomemory Performance of ITO/CS:GO/N

**Figure 8:**
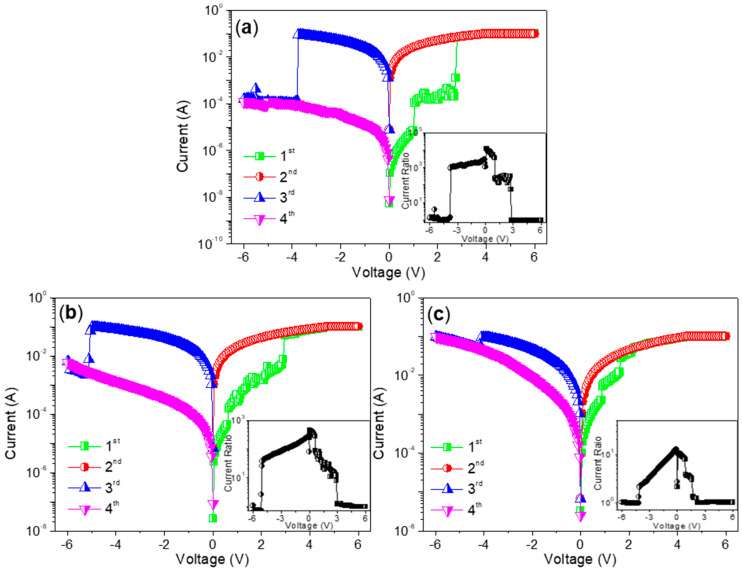
*I*–*V* characteristics of the multi-bit biomemory behaviors for ITO/CS:GO/Ni concerning distinct chemical component ratio of CS:GO naoncomposite (**a**) 2:1, (**b**) 5:1, and (**c**) 10:1 (*w*/*w*), respectively. Inset: the current ratio dependent on the applied bias.

*I*–*V* characteristics of ITO/CS:GO(2:1)/Ni were carried out at room temperature and measured with limited current compliance up to 0.1 A (Figure 8a). That the current abruptly increased at a SET voltage of 1 V (*V*_SET1_) leaded to the device turning from HRS to an intermediate resistance state (IRS), whose process was denoted as SET1. It suddenly gave rise to a second current growth for the device switching from IRS to LRS when the applied bias rose up to 2.75 V (*V*_SET2_). That could be regarded as a SET2 process. Furthermore, the performance of the biomemory devices was preserved in LRS during the voltage sweeping from 0 V to 6 V again. The current, when the negative bias was swept from 0 V to −6 V, dramatically decreased at a bias voltage of −3.8 V (*V*_RESET_). A transition from LRS to HRS for the device was displayed as a RESET process. In addition, the current proportion of HRS, IRS, and LRS (*I*_HRS_/*I*_IRS_/*I*_LRS_) was estimated to be about 1:10^2^:10^3^ during the biomemory process.

**Figure 9:**
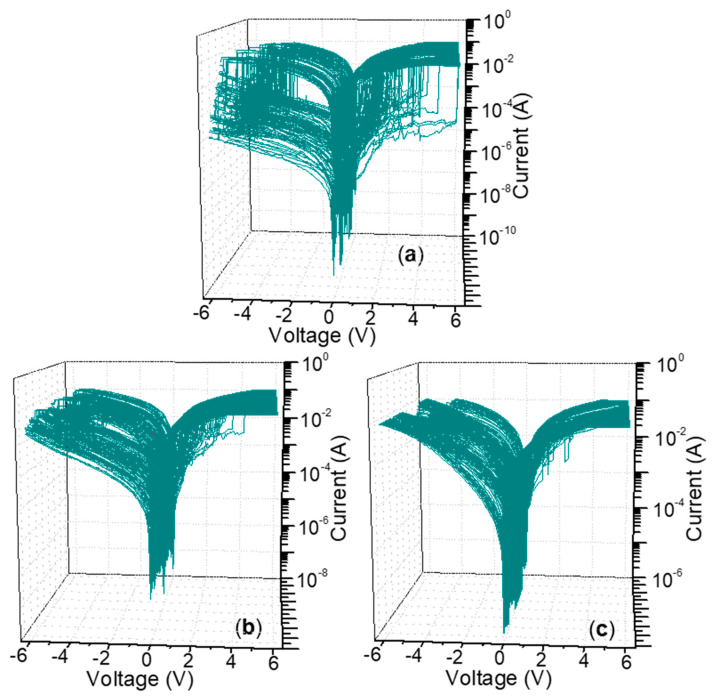
(**a**–**c**) Consecutive *I*–*V* cycles of the electrically ternary biomemory behaviors for ITO/CS:GO/Ni with weight ratio of chemical component 2:1, 5:1, and 10:1 *w*/*w*, respectively.

**Figure 10:**
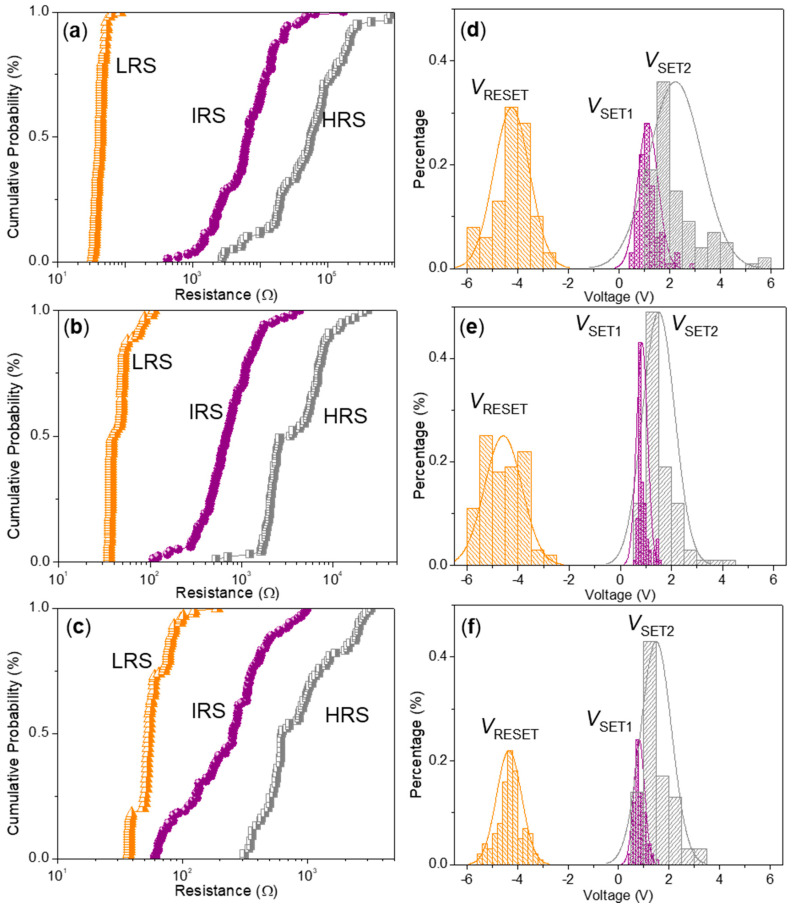
Cycle-to-cycle statistical distribution of (**a**–**c**) the resistance in HRS, IRS, and LRS (*R*_HLS_, *R*_ILS_, and *R*_LRS_), and (**d**–**f**) the set and reset voltage (*V*_SET1_, *V*_SET2_, and *V*_RESET_) for ITO/CS:GO/Ni with weight ratio of chemical component 2:1, 5:1, and 10:1 *w*/*w*, respectively.

**Figure 11:**
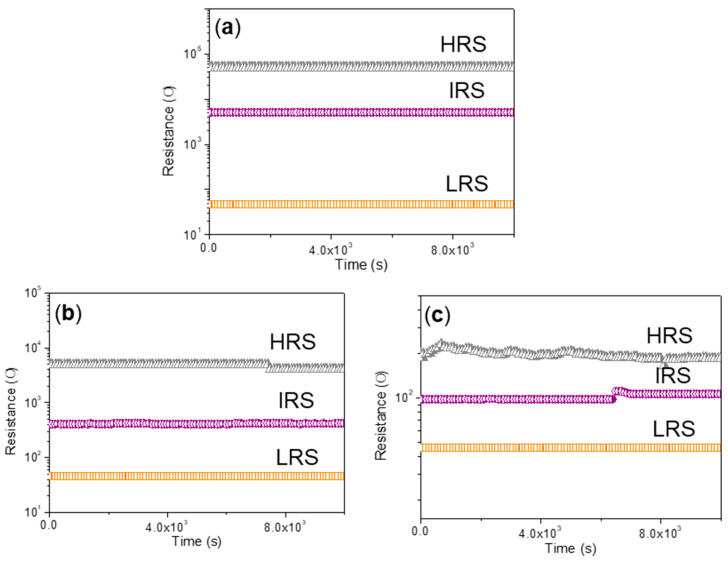
Retention characteristics of ITO/CS:GO/Ni devices based on CS-based blends with (**a**) 2:1, (**b**) 5:1, and (**c**) 10:1 (*w*/*w*). The resistance value was read at −0.1 V during the retention time and was investigated under ambient condition.

For ITO/CS:GO(5:1)/Ni (Figure 8b), *I*_HRS_/*I*_IRS_/*I*_LRS_ was calculated to be nearly 1:10:10^2^ when *V*_SET1_, *V*_SET2_, and *V*_RESET_ were 0.65 V, 2.95 V, and −5.1 V, respectively. As for ITO/CS:GO(10:1)/Ni (Figure 8c), it also witnessed ternary resistive-switching behavior with the current ratio *I*_HRS_/*I*_IRS_/*I*_LRS_ only close to 1:3:9, *V*_SET1_ = 0.9 V, *V*_SET2_ = 1.65 V, *V*_RESET_ = −4.2 V. Obviously, the aforementioned devices showed ternary resistive-switching behaviors for multi-bit biomemory application. A total of 100 consecutive cycles for ITO/CS:GO(2:1)/Ni, ITO/CS:GO(5:1)/Ni, and ITO/CS:GO(10:1)/Ni were carried out under ambient condition (Figure 9a–c). In this research, three different CS:GO nanocomposite compositions (2:1, 5:1, and 10:1, *w*/*w*) were taken into account. Among them, the percentage of GO content is 33.3%, 16.7%, 9.1%, respectively. For extension on the discussion about the performance of the material as biomemory concerning the GO content, more information of cycle-to-cycle statistical distribution for the device in HRS, IRS, and LRS (Figure 10a–c and Table 2) were provided. It indicates that *R*_HRS_ and *R*_IRS_ increase with incremental GO content in CS:GO nanocomposites. Figure 10d–f presents the cycle-to-cycle statistical distribution of the switching voltage *V*_SET1_, *V*_SET2_, and *V*_RESET_. As indicated by the normal fitting results, the biomemory devices based on CS:GO nanocomposites exhibited a relative concentrated distribution of SET1, SET2, and RESET voltages. The concentrated value for ITO/CS:GO(2:1)/Ni was separately 1.2 V, 2.2 V, and −4.2 V when that for ITO/CS:GO(5:1)/Ni was separately 0.8 V, 1.5 V, and −4.6 V. Moreover, the centrality bias for ITO/CS:GO(10:1)/Ni was 0.8 V, 1.5 V, and −4.4 V, respectively. Control experiments were elaborately conducted when an active CS layer was sandwiched between ITO and Ni electrodes (ITO/CS/Ni). No hysteresis effects were observed in *I*–*V* characteristics of ITO/CS/Ni. It was evident that the ternary resistive-switching behaviors of CS:GO nanocomposites were originated from GO. Under ambient condition, retention tests were performed on ITO/CS:GO/Ni in LRS, IRS, and HRS at a reading voltage of −0.1 V (Figure 11). During the retention time of 10^4^ s, the resistance of CS:GO nanocomposite films in LRS, IRS, and HRS (*R*_LRS_, *R*_IRS_, and *R*_HRS_) continuously kept stable without any degradation. These biomemory devices demonstrated the capacity of multi-bit nonvolatile random access memory.

**Figure 12:**
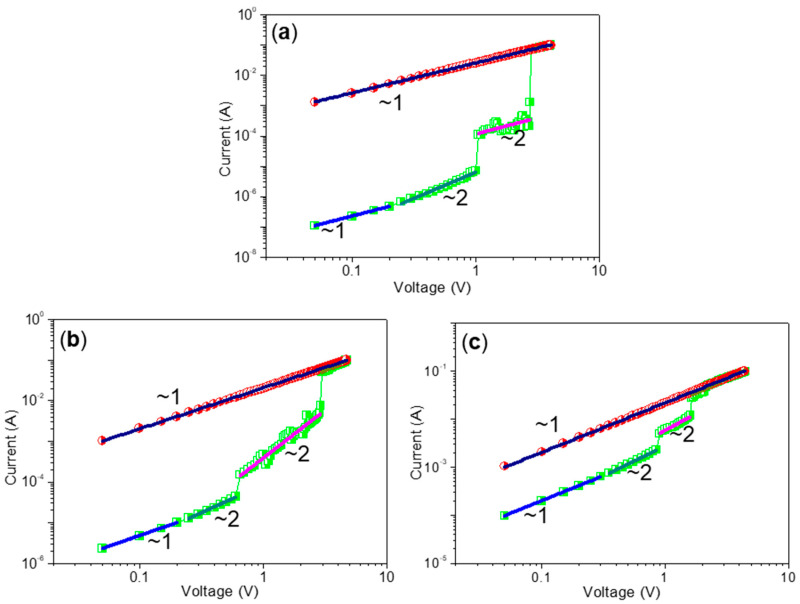
log*I*–log*V* curves with fitted conduction mechanism under positive (1st and 2nd sweep) DC sweeps in ITO/CS:GO/Ni devices with weight ratio of chemical component CS:GO (**a**) 2:1, (**b**) 5:1, and (**c**) 10:1 *w*/*w*, respectively.

In an effort to further the conduction mechanism of the ternary resistive-switching behavior achieved from ITO/CS:GO/Ni devices, *I*–*V* profiles were plotted in a log–log scale (Figure 12). In the low voltage region of HRS, a linear fitting (slope of ~1) for the double logarithmic scale indicated an Ohmic conduction behavior. Nevertheless, the log–log curves following a quadratic nature were deviated to realize a higher slope region (~2) after increasing the applied voltage. Since CS is an insulator in the native state, the injected charge carriers were believed to be gradually captured in the trap centers of GO up to saturation. The log*I*–log*V* curve for LRS (a slope of 1) transparently showed an Ohmic conduction behavior. It was consistent with the formation of a conducting path in the device after the SET2 process. In general, the features in the trap controlled space charge limited conduction (SCLC) model followed the correlation *I* ∝ *V*^n^, where *V* was denoted as an applied bias between the two electrodes and *n* was denoted as a positive number. The aforementioned conclusions were further corroborated upon application of both Poole–Frenkel and Schottky emission mechanisms to fit the resistive-switching behavior. The plots of ln*I* ∝ *V* and ln*I* ∝ *V*^1/2^ before transition to Ohmic conduction were not linear and showed parabolic behavior. It supported that the ternary resistive-switching mechanism was based on the SCLC mechanism with GO acting as trap centers in the CS:GO matrix.

## 4. Conclusions

Unlike state-of-the-art Si-based devices, CS-based nanocomposites blended with GO by means of the solution-processable method were seen as the biodegradable layer for multi-bit biomemory. It had a remarkable potential for nonvolatile biomemory, with a large current proportionality *I*_HRS_/*I*_IRS_/*I*_LRS_ (1:10^2^:10^3^), excellent cycling performance (>100 cycles), and long data retention characteristics (>10^4^ s) at ambient temperature. By combining the nature of CS and the carrier transport information extracted from double-logarithmic *I*–*V* characteristics, GO acting as trapping sites in the CS:GO nanocomposite layer might be responsible for the ternary biomemory mechanism. This paper shows the feasibility to fabricate multi-bit biomemory devices based on CS:GO nanocomposite, which paves the way for next-generation green data-storage devices and biointegrated electronics.

## Figures and Tables

**Table 1:** Summary results of Raman analysis for GO and CS:GO nanocomposites.

	D Band (cm^−1^)	G Band (cm^−1^)	2D Band (cm^−1^)	D + G Band (cm^−1^)	*I*_D_/*I*_G_	*I*_2D_ */I*_G_
GO	1347	1591	2703	2916	0.923	0.0567
CS:GO (2:1 *w*/*w*)	1351	1602	2697	2944	0.969	0.135
CS:GO (5:1 *w*/*w*)	1351	1604	2688	2942	0.954	0.0667
CS:GO (10:1 *w*/*w*)	1354	1607	2690	2943	0.953	0.0632

**Table 2:** Data analyses for ITO/CS:GO/Ni concerning the mean and deviation values of the resistance in HRS, IRS, and LRS (*R*_HLS_, *R*_ILS_, and *R*_LRS_).

Nanocomposites	*R*_HRS_ (Ω)		*R*_IRS_ (Ω)		*R*_LRS_ (Ω)	
	mean	std	mean	std	mean	std
CS:GO (2:1)	1.151 × 10^5^	1.882 × 10^5^	1.170 × 10^4^	1.977 × 10^4^	43.492	7.402
CS:GO (5:1)	5.188 × 10^3^	4.433 × 10^3^	910.115	757.711	47.429	15.935
CS:GO (10:1)	1.0582 × 10^3^	791.0759	296.611	219.764	60.310	22.291
